# Condylar reshaping in a TMD patient after ortho-prosthetic treatment: A 20-year follow-up case report

**DOI:** 10.4317/jced.59262

**Published:** 2022-02-01

**Authors:** Raffaele Schiavoni, Rossella Contrafatto, Camilla Grenga, Biagio Pacella

**Affiliations:** 1 MD, DDS. private practice; 2 DDS. private practice

## Abstract

Temporomandibular disorders (TMDs) involve different conditions with similar symptoms and signs affecting the temporomandibular joints (TMJs), the muscles of mastication, or both. The symptoms and signs of TMDs are several: pain and tenderness in and around the TMJs and muscles of mastication, limitation and lack of coordination in jaw movement, joint sounds – clicking and/or crepitus, episodes of closed lock or open lock, headaches. In the TMD, degenerative joint disease (DJD), also called osteoarthritis (OA), may be a local condition or part of a systemic disease. According to the Research Diagnostic Criteria for Temporomandibular Disorders RDC/TMD, OA involves the presence of reported symptoms and clinical signs. As regards the possible causes, the literature indicates that mechanical load may act as a trigger factor for a various degenerative modification resulting in con-dylar resorption when host remodeling capacity is lost. Although the role of occlusion in the etiology of TMD is not absolutely assessed, occlusal interferences could affect TMD. The aim of this case report is to present the history over time (20 years follow-up) of a female adult patient with osteoarthritis of TMJs, who showed an adaptative reshaping of the condyles and to consider the possible relation with orthodontic treatment and occlusal rehabilitation.

** Key words:**TMD, osteoarthritis, condylar reshaping, adult treatment.

## Introduction

In 1934, Costen reported a syndrome in which he hypothesized that occlusal modification may produce effects on the Temporomandibular Joint (TMJ).

30 years later, Thompson, an orthodontist, explained that some malocclusions could cause a condylar displacement. Consequently, the malocclusion correction could have improved temporomandibular disorders symptoms (1).

However, recent studies concluded that there is no correlation between TMDs and dental malocclusion and there is no scientific evidence that orthodontic treatment can be considered neither etiology nor cure for TMD symptoms (2,3).

Among TMD, one of the most frequent is anterior disc displacement (ADD),whose symptoms and signs are joint sounds and clicking, joint pain or tenderness, mandibular range of motion, masticatory difficulty, etc.

Several imaging studies have shown that continued disc deformity, flattening and deformation of the articular eminence and regression of the condylar size (Degenerative joint disease - DJD) are likely to happen in patients with internal derangement (ID) and anterior disk displacement (4).

It has been estimated DJD has a prevalence ranging from 8% to 35% (5). The percentage grows up to 60% in individuals with disc displacement without reduction (6).

In the TMD, degenerative joint disease (DJD), also cal-led osteoarthritis (OA), may be a local condition or part of a systemic disease. The use of instrumental investigations, such as MRI and CT Cone Beam, is necessary to highlight bone changes and to confirm the diagnosis.

As regards the possible causes, the literature indicates that mechanical load may act as a trigger factor for a various degenerative modification resulting in condylar resorption when host remodeling capacity is lost (7).

OA leads to malocclusion characterized by a mandibular backward rotation, distal molar occlusion with increased overjet due to the reduction of the joint space and probably caused by anterior or medio-anterior displacement of the disc. Eventually, although the role of occlusion in the etiology of TMD is not absolutely assessed, occlusal interferences could affect TMD (8).

The aim of this case report is to present the history over time (20 years follow-up) of a female adult patient with osteoarthritis of TMJs, who showed an adaptative reshaping of the condyles and to consider the possible relation with orthodontic treatment and occlusal rehabilitation.

## Case Report

In 1996 Stefania, a 31 year old woman, comes to our observation with TMD symptoms: bilateral click during opening and closing movements, neck pain, headaches, pain when chewing.

**Figure 1:**
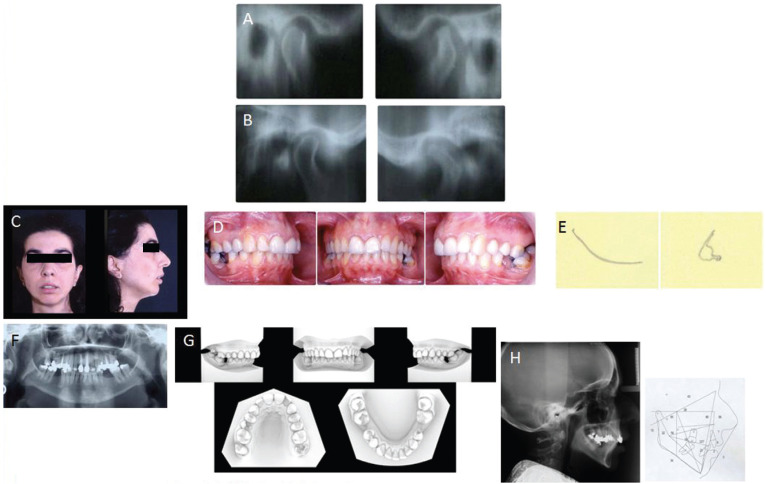
Pretreatment records: A) Initial bilateral TMJ tomography showing posterior bilateral condyle displacement. B) Pretreatment bilateral TMJ tomography. C,D) Pretreatment facial and intramural views. Due to the reduced mouth opening consequent to the joint pain, it was not possible to carry out routine occlusal photos. E) Pretreatment axiographic tracing of opening and closing movements. F) Pretreatment panoramic. G) Pretreatment digitized dental casts. H) Pretreatment lateral radiograph and cephalometric tracing and lateral radiograph.

Serial tomography, the chosen examination during that time, is prescribed. The diagnosis is “bilateral posterior condylar displacement” (Fig. 1). Moreover, images show a regressive remodeling of the left condyle with flattening or concavity of the posterior wall. ADD was diagnosed based on symptoms, because in 1996 MRI was not yet available. At the time the patient rejects any kind of therapeutic approach proposed.

She comes back to our observation four years later after several open and closed lock episodes, the last of which requested night-time unlocking intervention in public health facilities.

The patient reports that in the last five years she has continuously been having TMJ pain during mandibular movements, occasional spontaneous pain and frequent headaches.

Intraoral examination

Occlusal examination shows:

1. bilateral class II occlusal relationship, more markedly on the left side;

2. absence of 3.6, 4.6 and 1.7;

3. deviation of lower midline to the left;

4. 1.8 totally inclined medially;

5. 3.7 and 4.7 significantly tipped to the first molar extraction site;

6. Reduction of the posterior vertical dimension consequent to tooth decay of 2.7.

2.7, severely decayed, is extracted. Due to the reduced mouth opening consequent to the joint pain, it was not possible to carry out routine occlusal photos (Fig. 1).

Extraoral examination

Facial and cephalometric analysis (Quick Ceph Systems, San Diego, CA) shows class II hyperdivergent pattern. All the vertical parameters indicate this kind of aspect: FMA 36.6°, SN-GoGn 40°, ANS-PNS /GoGn 40°; Wits appraisal is 5.7 mm and OVJ is 5.4 mm. The position of lower incisors on the mandibular plane is 95°.

Radiographic examination

Radiographic observations carried out by means of serial tomography shows, in relation to the radiographic images performed four years earlier, a marked “kinking” of the neck of left condyle. The left side was the most occlusally damaged due to the loss of 2.7.

No modifications to the right joint have occurred in the past years.

During opening and closing movements lower jaw fol-lows an S-shaped path typical for ADD with disc recapture.

Axiographic tracing, done with SAM system (PrazionstechnikGmbH, Munich, Germany), shows on the left side the typical path of ID.

**Figure 2:**
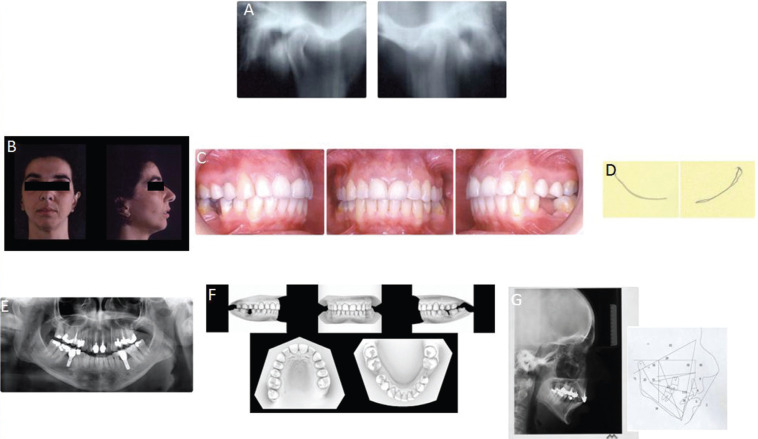
Posttreatment records. A) Post-treatment tomography shows further reshaping and repositioning of both condyles. B,C) Post-treatment facial and intramural views. D) Post-treatment axiographic tracing of opening and closing movements. E) Post-treatment panoramic. F) Post-treatment digitized dental casts. G) Post-treatment lateral radiograph and cephalometric tracing.

**Figure 3:**
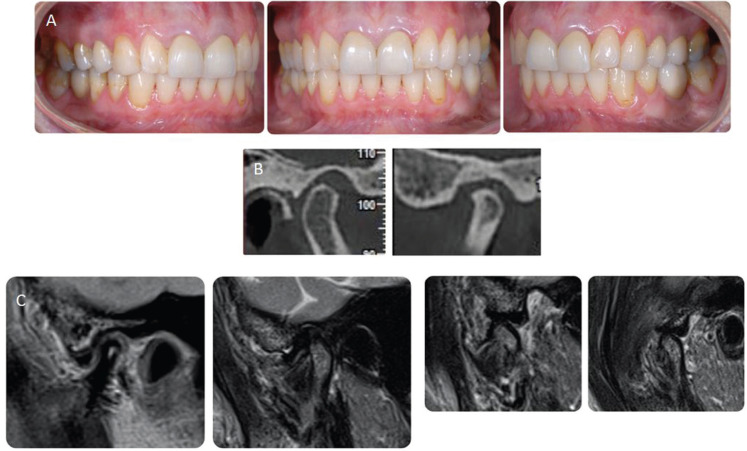
20 years follow up records. A) Intraoral views 20 years after the end of treatment showing good stability of occlusion. B) Serial tomography reconstruction by CBCT 20 years after the treatment showing reshaping of condyles. C) MRI 20 years treatment closed and open mouth shows anterior disc displacement bilaterally.

All these diagnostic findings are in accordance, as well as with the diagnosis of bilateral posterior condylar displacement, with the presence of an osteoarthritic degeneration of the joint heads, especially on the left side (remodelling and hypertrophy of bone are major features of OA) (Figs. 2,3).

The concavity of posterior slope of eminence could increase probability of anterior disc displacement and the unfavorable position 3.7 and 4.7 could be compared to class III forces’ vector that can influence functional changes and remodeling in shape of the articular heads.

-Treatment objectives

The main goal is relieving the dysfunctional symptomatology. The proposed therapy involves orthodontic treatment whose aim is the correction of the malocclusion and the reconstitution of a functional lower occlusal Table that should generate an “unloading” of the joints through counterclockwise rotation of the lower jaw.

To achieve an occlusal balance, first we plan to restore the lost posterior vertical dimension and provide posterior stability in intercuspal position (ICP) with no deflective contacts during arc of closure; then we arrange to get a cuspid and anterior guidance to avoid any interference during protrusive and lateral movements.

Considering the patient’s age and the disorder period, the patient is informed that this therapy will not necessarily resolve the symptoms induced by TMDs.

-Treatment alternatives

Another treatment which could be proposed, since pain is the main chief complaint together with several episodes of closed lock and considering the patient’s willingness to not undertake any drug therapy, is the use of anterior repositioning appliance (ARA) to be worn at night and, if necessary, also during the day (9).

The patient refuses this kind of treatment since speech impediment is incompatible with her work.

-Treatment plan

The accepted therapeutic program includes:

I. uprighting of 3.7 and 4.7 to create space for two fixtures in region 3.6, 4.6;

II. levelling of curve of Spee;

III. repositioning of the mandible on the horizontal plane to achieve first-class canine on the left, midline correction and a more centered condylar position;

IV. prosthetic finalization.

-Treatment progress

Orthodontic treatment has been done with preadjusted appliance (MBTprescription 3M Unitek, Monrovia, CA, USA), and it lasted 30 months.

After eight months, the right second lower molar uprighting was carried out and the most posterior teeth become the occlusal pivot allowing spontaneous relocation of the condyles into the seated position (10).

With the new occlusion the mandible rotates clockwise as the condyles sit further forward and higher, resulting in an anterior open bite and a more severe dento-skeletal class II relationship with a steeper mandibular plane.

At this time all TMD symptoms disappeared.

Then a left monolateral Forsus appliance (3 M Unitek Corp., St. Paul, MN, USA) was applied to reposition the mandible in the horizontal plane and to facilitate on the left side the achievement of class I occlusal relationship.

-Treatment results

Class I cuspid relationship was achieved on both sides. Overjet and overbite were normal, and a sTable posterior occlusion was present (OVJ changed from 5,4 mm to 3 mm and OVB from 3,1 mm to 1 mm).

Cephalometry post treatment values do not show significant changes except for the position of lower incisors, probably due to the Forsus device. Proclination of lower incisor from 95° to 104° (IMPA) allowed the correction of OVJ and at the same time the achievement of class I cuspid relationship. Wits appraisal changed from 5,7 to 3,2. Inter-incisive angle changed from 133° to 121°. The lower midline was also almost centered and up righting of second molar was totally performed.

At the end of the orthodontic treatment, fixtures were positioned in the lower jaw and then prosthetic finalization is carried out.

Serial tomography showed a reshaping and a repositioning of both condyles, especially on the surface of the left condyle it was observed a positive adaptive remodeling. Furthermore, uniform and wider joint spaces were obtained. Axiographic tracings shows normal path of opening and closing movement (Fig. 2).

-Follow-up

Twenty years posttreatment, the patient is in good general condition and no signs of TMJ pain or dysfunction were present. The anamnestic investigation, done during the follow up visit, detects no pathological events during the past years.

The patient shows a total disappearance of dysfunctional symptoms and the clinical exam reveals a sTable occlusion. She willingly undergoes the new diagnostic tests required (MRI/CBCT) to monitor the evolution of the remodeling of the joints.

No periodontal problems in the lower front teeth have been highlighted.

CBCT does not show any phenomenon of condylar erosion but rather the maintenance of the shape and position reached at the end of the orthodontic-prosthetic rehabilitation. The intra-articular space remained physiological as well as the condyle-glenoid fossa relationship. MRI shows the reduction of disc dislocation during masticatory function although it remains slightly anteriorized (Fig. 3).

## Discussion

The TMJs undergo continuous remodeling through our whole lifetime due to both parafunctions and the mechanical loading to which they are subjected during the masticatory function. OA, as defined by the Diagnostic Criteria for Temporomandibular Disorders, is a degenerative disorder involving the joints characterized by deterioration of tissues with concomitant osseous changes in the condyle and/or articular eminence (11).

OA may culminate in a modification of the physiological condylar form and structure. If the modification is unilateral can result in a mandibular asymmetry/deviation with canting of occlusal plan; if the modification is bilateral a mandibular retrusion with possible opening anterior bite can occur.

Conventional management goals for degenerative joint disease or OA include relieving signs and symptoms, restoring normal function, and preventing further joint damage (12).

If the normal masticatory load leads to a physiological remodeling of the joint heads, the biomechanical overload has been commonly assumed to be an important predisposing factor for degenerative changes in TMJs (5).

The pathological mechanism due to excessive or unbalanced mechanical loading could be the increase in intra-articular pressure leading to transient hypoxia with consequent cortical destruction and general degradation of the TMJ structures (13).

The possibility of regenerative condylar remodeling in terms of both form and structure exists (14). The literature suggests that this is possible thanks to the decrease in mechanical stress on the joints and/or improved disc-condyle relationships (15).

In our case study, we hypothesize that orthodontic treatment and subsequent prosthetic rehabilitation may have favored the forward-downward position of the condyle and caused biomechanical discharge of the TMJ facilitating regenerative remodeling of the condylar bone.

The patient axiographic tracings before treatment showed a serious deformation of the left condyle with anterior disk displacement without reduction. Post orthodontic treatment checks showed acondylar morphology improvement and the disappearance of the majority of TMD symptoms.

We speculate that the condylar morphology improvement, visible through the CBCT tomograms, may be the result of an adaptive change or functional remodeling, due to orthodontic treatment and reduction of the biomechanical load.

The condyles showed rotational repositioning with increased joint space in the superior and anterior areas and condylar position adjustments could be linked to the occlusal improvement. A better condyle-fossa relationship could lead to positive condylar reshaping. Resuming: orthodontic treatment, the subsequent prosthetic rehabilitation and the increase of the posterior vertical dimension were planned to curtail the overloading.

Through MRI we notice that the disc is recaptured by the patient in opening even if it remains slightly anteriorized. As described in another case report,condylar morphology improvement could be due to a better condyle-fossa relationship and a balanced occlusion.

Finally, it is important to point out that when treating patients with TMD orthodontically, the ever-changing symptoms and occlusion caused by an unstable condylar position prevent reliable criteria for orthodontic treatment planning.

## Conclusions

Proper relocation of the mandible through orthodontic treatment could positively activate the bone remodeling process and lead to repositioning and reshaping of the condyle with the consequent disappearance of the painful symptoms.

Among the various attentions to be placed in planning the orthodontic treatment, an absolutely primary role must be covered by the attention paid to preventing an overload of the temporomandibular joints.
